# Chromoendoscopy in magnetically guided capsule endoscopy

**DOI:** 10.1186/1475-925X-12-52

**Published:** 2013-06-11

**Authors:** Philip W Mewes, Stefan Foertsch, Aleksandar Lj Juloski, Elli Angelopoulou, Stefan K Goelder, Dirk Guldi, Joachim Hornegger, Helmut Messmann

**Affiliations:** 1 Pattern Recognition Lab, University of Erlangen-Nuremberg, Martensstrasse 3, Erlangen Germany; 2 Siemens AG, Healthcare Sector, Allee Am Roethelheimpark 2, Erlangen 91052, Germany; 3 Department of Chemistry and Pharmacy, Friedrich-Alexander University, Egerlandstr. 3, Erlangen 91058, Germany; 4 Medizinische Klinik III, Klinikum Augsburg, Stenglinstr.2, Augsburg 86156, Germany

**Keywords:** Methylene blue, Indigo carmine, Staining

## Abstract

**Background:**

Diagnosis of intestinal metaplasia and dysplasia via conventional endoscopy is characterized by low interobserver agreement and poor correlation with histopathologic findings. Chromoendoscopy significantly enhances the visibility of mucosa irregularities, like metaplasia and dysplasia mucosa. Magnetically guided capsule endoscopy (MGCE) offers an alternative technology for upper GI examination. We expect the difficulties of diagnosis of neoplasm in conventional endoscopy to transfer to MGCE. Thus, we aim to chart a path for the application of chromoendoscopy on MGCE via an ex-vivo animal study.

**Methods:**

We propose a modified preparation protocol which adds a staining step to the existing MGCE preparation protocol. An optimal staining concentration is quantitatively determined for different stain types and pathologies. To that end 190 pig stomach tissue samples with and without lesion imitations were stained with different dye concentrations. Quantitative visual criteria are introduced to measure the quality of the staining with respect to mucosa and lesion visibility. Thusly determined optimal concentrations are tested in an ex-vivo pig stomach experiment under magnetic guidance of an endoscopic capsule with the modified protocol.

**Results:**

We found that the proposed protocol modification does not impact the visibility in the stomach or steerability of the endoscopy capsule. An average optimal staining concentration for the proposed protocol was found at 0.4% for Methylene blue and Indigo carmine. The lesion visibility is improved using the previously obtained optimal dye concentration.

**Conclusions:**

We conclude that chromoendoscopy may be applied in MGCE and improves mucosa and lesion visibility. Systematic evaluation provides important information on appropriate staining concentration. However, further animal and human in-vivo studies are necessary.

## Background

Although incidence and mortality are decreasing, gastric cancer with 738.000 cases worldwide in 2008 is the 2nd most lethal digestive neoplasm in the world [1]. Intestinal metaplasia and dysplasia are precursors of cancer [2]. The identification of these lesions and follow-up of afflicted patients could lead to early diagnosis and treatment, and thus enhance the survival of the patient [3,4]. Esophagogastroduodenoscopy (EGD) is the most common procedure for diagnosis and treatment. However, for the detection of metaplasia and dysplasia conventional EGD is characterized by low interobserver agreement and poor correlation with histopathologic findings [5,6].

Various techniques are available for enhancing and highlighting mucosa irregularities and for increasing the visibility of structures which lie under the surface of the mucosa. The most important methods include narrow band imaging, confocal laser endomicroscopy, magnification endoscopy, optical coherence tomography and chromoendoscopy [7-10]. These techniques have often been compared against each other, or in combination in terms of their impact in diagnostic accuracy (e.g. in [11,12]). However a substantial difference between chromoendoscopy and all competing techniques lies in the lack for additional hardware. Chromoendoscopy requires no modification of the hardware of the imaging system itself.

Furthermore, chromoendoscopy in EGD and colonoscopy has been shown to significantly enhance the visibility of mucosa irregularities, like metaplasia and dysplasia [13]. Chromoendoscopy consists of the topical application of different stains to improve tissue visibility, localization and characterization for the purpose of better diagnosis. Chromoendoscopy usually consists of four steps for absorptive stains and three steps for contrast stains: (1) Application of an acid solution to dissolve gastric mucus, (2) local application of a stain, (3) (only for absorptive stains) washing of the respective region with water and (4) visual inspection of the stained regions for diagnostic purposes. In (1)-(3) the application of dye is performed locally using the working channel of the endoscope and different spray catheters (direct method) under the visual guidance of the endoscope. For colonoscopy the passive application of stain with a dye-powder filled capsule has also been reported [14,15]. In this procedure a capsule with dye powder is given to the patient after administration of a bowel cleansing solution such as PEG. Between the dye administration and the colonoscopy examination a waiting time is needed. Application of the dye in the morning and examination in the afternoon was reported as a sufficiently large time span [14]. Though the procedure was found to be feasible, difficulties have been reported due to the inhomogeneous application of the stain [16]. The oral application of dye for examination of the stomach without using a spray catheter (indirect method) has been described in [15,17].

Recently, different approaches for magnetically guided capsule endoscopes (MGCE) for gastric and small bowel examinations were presented [18-23]. In a clinical study on humans, MGCE showed the feasibility of gastric exploration with a guided capsule endoscope [18,19]. In this specific study the stomach was filled with water and the capsule was navigated from the outside using an external magnetic field. An operator could control the motion of the capsule during the examination using feedback from real-time gastric imaging provided by two capsule camera sensors. Hence, he could obtain a sufficient number of stomach-surface images with diagnostic value.

We expect that known difficulties in the diagnosis of neoplasia, regarding interobserver agreement in conventional endoscopy, transfer to MGCE. MGCE could, thus, benefit from chromoendoscopy in the same way classic endoscopy does. However, compared to the direct application of stain in EGD and most colonoscopy procedures, in MGCE only indirect application is possible. No acid preparation and washing of the gastric mucosa is possible. Furthermore, the water in which the capsule is maneuvered, must not be stained at levels which reduce the overall visibility. Competing methods such as narrow-band imaging in the diagnosis of colorectal neoplasia are difficult to integrate in a capsule endoscope [11]. Furthermore, as in many endoscopic techniques the exact impact of chromoendoscopy and the technical details have not yet been established [24,25]. For example, in literature ([5,26,27]) one can find three different concentrations of Methylene blue dye and application times for the examination of gastric neoplasm. The search for an optimal concentration for a staining procedure in animal and human trials has been reported a few times, but without the support of a thorough analysis. In [28] an optimal staining concentration for simultaneous confocal laser endomicroscopy and chromoendoscopy with Cresyl violet is assessed in mices, but without an objective criterion. In [29] an optimal staining concentration for endocytoscopy was accessed in an ex-vivo animal study in which freshly resected porcine esophagus, stomach, and colon were examined. Image contrast and staining status were evaluated by experts for each organ to determine the best concentration. Results were transferred to resected human organs. The problem of a missing systematic study for classic chromoendoscopy in the stomach transfers to MGCE and becomes more severe through the challenges of the indirect application of stain.

In this paper we evaluate the possible application of chromoendoscopy to MGCE in an ex-vivo animal study. **First**, we propose a modification to the MGCE preparation protocol in order to incorporate a staining procedure for chromoendoscopy. **Second**, we present a method to systematically assess an optimal concentration of dye for the proposed protocol modification. The optimization is conducted in experiments using pig stomach tissue samples and with respect to the best visibility of tissue of different histological or pathological nature. **Third**, we transfer these results to an ex-vivo pig stomach experiment under magnetic guidance of a capsule endoscope. These experiments should determine: a) the overall under-water visibility after the proposed passive staining protocol; and b) the mucosa and lesion visibility with the optimized dye concentration.

The guidance magnet is technically similar to the one used for the human study [18,19]. All navigation functions of that study are also available in our setup. The system is a joint development of SIEMENS Healthcare and Olympus Medical Systems Corp. Its main components are: (1) A guidance magnet that consists of a set of electromagnetic coils defining a working volume and enabling the operator to control a capsule endoscope with 5 degrees of freedom (DOF). The magnetic flux density has a maximum of 100 millitesla. (2) A capsule endoscope of 31mm length manufactured by Olympus Medical Systems Corp. with a build-in permanent magnet and two CCD cameras each transmitting 2 frames per second in real time to an external receiver attached to the patient’s body. (3) A display showing the capsule images to the operator. (4) A set of joysticks allowing the operator to maneuver the capsule inside the stomach. The orientation of an electromagnetic (EM) field orients the capsule in the stomach. The EM field together with an EM gradient field generate forces on the capsule endoscope of less than 1 millinewton. These are sufficient for translational movements. More details about the hardware and software design of the guidance magnet can be found in [30]. The guidance of the capsule is performed based on real-time imaging provided by the capsule endoscope inside the pig stomach.

In [31] a pig stomach study was presented to improve mucosa visibility in MGCE using Methylene blue. This study was limited to only a few cases and a single dye. The magnetic steerability was only simulated using a plastic support and there was no systematic evaluation of an optimized dye concentration prior to the experiments. Whereas in this paper the magnetic steerability is achieved with a capsule guidance magnet. The study is conducted with a large number of pig stomachs and leads to a systematic assessment of two optimal dye types.

## Methods

### Modification of preparation protocol for MGCE

The established preparation protocol used for the existing human MGCE study with 43 patients consists of three administrations of tap water prior to the examination:[18,19]

#### *Existing MGCE preparation protocol*

E.1 500 mL of clear water at room temperature one hour and 15 minutes prior to the examination and after overnight fasting.

E.2 400 mL of clear water at room temperature 15 minutes prior to the examination followed by light exercises.

E.3 400 mL of clear water at near body temperature, immediately before the examination.

All applications are given orally. Steps E.1 and E.2 are primarily intended for cleaning the stomach. Step E.3 aims at expanding the stomach in order to obtain enough space for the capsule to be maneuvered and for complete visibility of the stomach mucosa without gastric folds overlapping each other and eventually hiding relevant mucosa parts. Step E.3 cannot be modified since it is crucial for the guidance of the capsule inside the stomach. We expect the water to be stained to such an extent that the general visibility is reduced when the stain is directly applied prior to E.3. Hence, we propose to fit a staining step between steps E.1 and E.2 of the existing preparation protocol. In order to conduct further experiments with animal phantoms the following protocol is adopted for pig stomachs:

#### *Modified MGCE preparation protocol for pig stomachs*

M.1 2000 mL of clear water at room temperature that is emptied out of the stomach immediately after administration. The purpose of this step is still the cleaning of the stomach from mucus and/or remaining food.

M.2 100 mL of dye, followed by 5 minutes of massage and kneading the stomach to simulate peristaltic movement, followed by emptying the stomach from the dye. This step applies the dye to the stomach walls. Massage and kneading the stomach simulates peristaltic motion and is performed under the assumption that the dye would be naturally scattered over all anatomical areas of the stomach in the natural case. Simulating digestive peristalsis via massage a stomacher bag or a pulley system to create peristaltic motion in a mechanical stomach model was reported in [32,33].

M.3 500 mL of clear water at room temperature, which remains in the stomach for 5 minutes and is afterwards emptied out. This step is similar to E.2 of the standard MGCE procedure but this time it also evacuates the remaining dye.

M.4 2000 mL of clear water at near body temperature, immediately before the examination (same purpose as E.3).

All applications are performed through the esophagus and intend to simulate the oral application of water and dye. Using a combination of water, to expand the stomach, and dye at the same time was assumed to be incompatible with MGCE since the visibility of the capsule would deteriorate. The emptying is performed by gently squeezing the stomach and imitates the natural evacuation of stomach content into the small intestine. The amount of water for washing and expanding the stomach (step M.1 and M.4) was set to larger values due to the larger size of a pig’s stomach.

### Target lesion and lesion imitations

**Figure 1:**
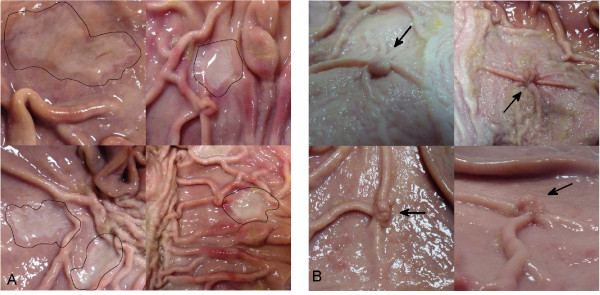
**Lesion imitations: Figure** 1**(A): Four examples of non-protruding slightly-depressed lesions (0-IIc) contoured with dark lines, Figure** 1**(B): Four examples of pseudopolyps (marked with arrows) to simulate a protruding lesion (0-lp).**

To assess the benefits of chromoendoscopy in MGCE the proposed method is tested on healthy mucosa and neoplastic lesion imitations. Lesions of possible early gastric cancer (EGC) are subdivided into 3 main categories: protruding (0-I), non-protruding and non-excavated (0-II), excavated (0-III) with each of these types having multiple sub-types [34]. Two lesion types are considered in order to evaluate the benefits of chromoendoscopy with the proposed modified protocol: pseudopolyps to simulate a protruding (0-lp) lesion and non-protruding slightly depressed lesions of type 0-IIc. In [35] a method is described to create a pseudopolyp using an esophageal variceal ligation device. We used a similar approach but employed a suture to perform a ligation of the stomach mucosa in order to create a pseudopolyp. For the simulation of 0-IIc non-protruding lesions 10% HCL solution was applied to the stomach mucosa for 15 seconds and washed off with tap water. Eight example images (four of 0-lp and four of 0-IIc) of the created lesion imitations are shown in Figure 1.

### Stains

During EGD or colonoscopy, different stains are used for chromoendoscopy. They are classified as absorptive, contrast, or reactive [24]. In our experiments we use an absorptive stain (Methylene blue) and a contrast stain (Indigo carmine). Methylene blue, is absorbed by specific cell types and highlights, therefore, through preferential absorption. Indigo carmine is not absorbent and highlights the mucosa by mechanically pooling in cervices between epithelial cells, fat or depressed lesions and other irregularities. The lesion imitations of type 0-IIc (early gastric cancer) are stained with indigo carmine as described in [36]. For the staining of fine details of the mucosa Methylene blue is used.

### Visual criteria of an optimal stain concentration

**Figure 2:**
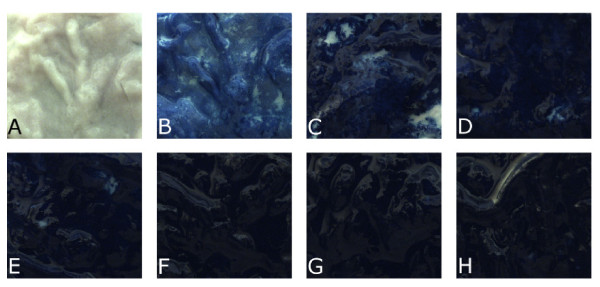
**Methylene blue staining: Pig stomach patches stemming from different stomachs stained with concentration from 0% to 1.4% Methylene blue.** (**A**): no staining, (**B**): 0.2%, (**C**):0.4%, (**D**):0.6%, (**E**):0.8%, (**F**):1%, (**G**):1.2%, (**H**):1.4%. Image of 2 x 2 cm pig stomach patches were cropped to 1.8 x 1.8 cm.

**Figure 3:**
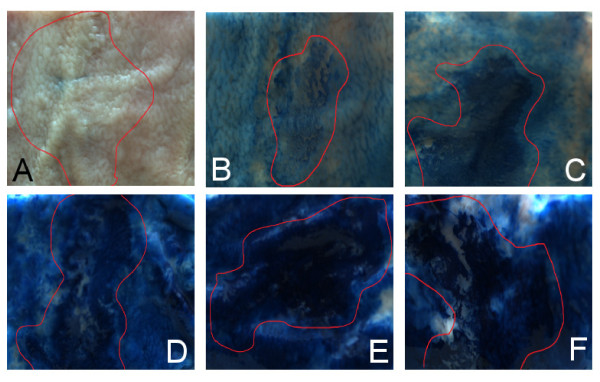
**Pig stomach patches with lesion imitation stained with different concentrations of Indigo carmine.** (**A**) no staining, (**B**) 0.2%, (**C**) 0.4%, (**D**) 0.6%, (**E**) 0.8%, (**F**) 1%. Non-protruding slightly-depressed lesions (0-IIc) are contoured with red outlines. Image of 2 x 2 cm pig stomach patches were cropped to 1.8 x 1.8 cm.

A staining procedure for chromoendoscopy in MGCE has additional challenges compared to classic chromoendoscopy involving a flexible endoscope: Local application of stain as used in EGD and colonoscopy is not possible in MGCE, neither is an acid preparation of the mucosa or washing of the gastric mucus after the application of stain. The optimal dye concentration is quantitatively and systematically assessed and further applied in the proposed modified protocol. To that end, a large number of images from pig stomach tissue samples is stained with different dye concentrations in order to determine an optimal staining concentration (See Figures 2 and 3). Since we found that simple visual inspection is not sufficient to accurately judge the optimal stain concentration, we defined two objective visual parameters to evaluate the concentration applied to healthy mucosa and 0-IIc lesion imitations:

1. **Lesion-to-background contrast:** Image contrast can be defined as the quantitative difference, in terms of color and intensity, between several neighboring spatial image regions or objects within an image. For endoscopic images contrast can be interpreted as the local qualitative difference in color and/or intensity between neighboring pathological and healthy tissue. An optimal stain concentration for the visualization of a lesion would, therefore, be defined by a concentration leading to a maximal contrast between pathologic and healthy mucosa. Chromoendoscopy aims to enhance this contrast as much as possible by the applied dye and resulting changes in the coloring of different mucosa types. We assign a numerical score *I*_*c *_which can informally be defined as the average contrast between lesion tissue and healthy tissue.

**Figure 4:**
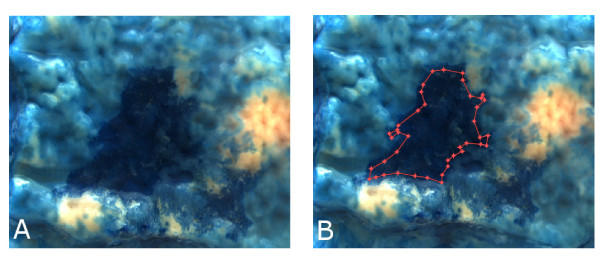
**Labeling method example: Figure** 2**(A): Without labels.** Figure 2(**B**): The segmented diseased tissue region is bounded by a red line. The points on the red line show the manually selected segmentation points.

For the computation of the contrast score *I*_*c *_two regions *R*_*l *_and *R*_*h *_are defined for the image region with lesions and the healthy tissue respectively. Since the contrast in an image can be described as the quantitative difference between different image regions. A measurement of the contrast *I*_*c *_between both regions can therefore be denotes as

(1) $$\begin{array}{l} I_{c}=\left|g\;(R_{l})-g\;(R_{h}),\;\right| \end{array}$$

where *g*(·) refers to the gray-scale conversion of the original color image. To compute such a contrast score, for each image the two regions *R*_*l *_and *R*_*h *_are manually chosen inside a region with lesions and the healthy tissue. This labeling process does not segment the exact border between healthy and diseased tissue. Manual segmentation is always subject to the expert. More notably an exact segmentation is relatively unimportant, compared to the overall contrast between two image regions. Hence an exact region segmentation is not necessary. Figure 4 shows an example of a manually segmented diseased tissue region. The value on a gray scale image lies between 0 and 255. Hence the score for the lesion-to-background contrast would also lie between these two values, where a small value would imply poor contrast and a high value would indicate an image with high contrast between lesion and background. In [29] such an approach was defined to determine the optimal contrast between the cytoplasm and the nuclei for endocytoscopy.

2. **Global texture variance:** Various texture measurements are known from computer vision in general and in particular from medical image processing for the purpose of automatic segmentation, classification or content based image retrieval [37,38].

Image texture can be described as a measurement of spatial arrangement and distribution of intensity and/or color in a image. Within these arrangements and distributions the variance can be measured. For images in endoscopy this can be the global quantitative difference in color and/or intensity between tissue of different histological or pathological nature and the intensity/color variance between these tissue areas. An image that exhibits high texture is an image with a considerable amount of distinguishable intensity/color variation. If at the same time the image exhibits a high variance between these image regions we translate this to an image with high texture variance. Chromoendoscopy aims to improve the global texture variance by highlighting tissue areas on the gastric mucosa with different characteristics as clearly as possible. An optimal dye would, hence, maximally enhance actually existing different tissue areas and would cause high variance in contrast between these different textures.

A popular feature to describe texture is the local binary pattern (lbp) [39]. This method has been widely applied in medical image processing and has, among other variations, been extended to quantify the global texture variance of an image [40]. We assign a numerical score *var*(*R*,*N*)_*g *_which can informally be defined as the contrast variance in image texture.

The computation of *var*(*R*,*N*)_*g *_is as follows: The basic principle of lbp is a discrete characterization of pixel neighborhoods. Each pixel in an image is assigned a value depending on how it relates to its neighboring pixels in term of intensity. The neighborhood is usually defined by two parameters, which are the number of pixels taken into account and the distance between the center pixel and its neighbors. In lbp the neighbors are arranged in a circle around a center pixel and therefore the distance can simply be defined by the radius of the circle. Each pixel on this circle is assigned the value 0, if its intensity is below the intensity of the central pixel and the value 1 if the intensity value is greater than that of the central pixel. For a center pixel with 8 neighbors that would result, for example, in an 8-digit binary number which leads to the original name of a linear binary pattern. A binary lbp pattern at a center pixel *p*_*c *_at the position (*c*_*x*_,*c*_*y*_) with its *N* neighbors pixels *p*_*n *_at an radius *R* can therefore be described as

(2) $$\begin{array}{lcr} \text{lbp}{\left(R,N\right)}_{\left(c_{x},c_{y}\right)}={\left(\overset{N}{\underset{i=1}{\sum }}b(p_{n}(i)-p_{c_{x},c_{y}})\times 2^{N}\right)}_{{\;(d\to b)}^{1}} & & \end{array}$$

where

(3) $$\begin{array}{l} b(x)=\left\{\begin{array}{ccc} 1 & \text{if} & x\geq 0\\ 0 & \text{if} & x<0 \end{array}\right) \end{array}$$

where *d*→*b* denotes the conversion of the previous term from a decimal to a binary number and the coordinates of each neighbors *p*_*n*_(*i*) can be calculated for a center pixel at the position (*c*_*x*_,*c*_*y*_) as $p_{n}{\left(i\right)}_{x}=c_{x}+R\times \text{cos}(2\times \pi \times \frac{i}{N})$ and $p_{n}{\left(i\right)}_{y}=c_{y}+R\times \text{sin}(2\times \pi \times \frac{i}{N})$. The decimal number $\text{lbp}{\left(R,N\right)}_{\left(c_{x},c_{y}\right)}$ needs then to be converted to a binary number in order to obtain the actual pattern. If the position of an neighbor pixel is not exactly in the center of the pixel grid, its value is bilinearly interpolated out of a 2×2 neighborhood. According to [41] Eq. 2 can be modified to measure the local texture variance. For a pixel *p*_*c *_at the position (*c*_*x*_,*c*_*y*_) with its *N* neighbors pixels *p*_*n *_at an radius *R* the texture variance is computed as

(4) $$\begin{array}{lcr} \text{var}{\left(R,N\right)}_{\left(c_{x},c_{y}\right)}=\frac{1}{N}\overset{N}{\underset{i=1}{\sum }}{\left(p_{n}(i)-\mu \right)}^{2}. & & \end{array}$$

with $\mu =\frac{1}{N}\overset{N}{\underset{i=1}{\sum }}p_{n}(i)$. To globally measure the texture variance for a entire image Eq. 4 needs to be applied to the entire image. Thus Eq. 4 is extended to

(5) $$\begin{array}{lcr} \text{var}{\left(R,N\right)}_{g}=\frac{1}{h\times v}\overset{h}{\underset{k=1}{\sum }}\overset{v}{\underset{l=1}{\sum }}\frac{1}{N}\overset{N}{\underset{i=1}{\sum }}{\left(p_{n}(i)-\mu \right)}^{2}. & & \end{array}$$

where the coordinates of each neighbors *p*_*n*_(*i*) for *k* and *l* are computed as $p_{n}{\left(i\right)}_{x}=k+R\times \text{cos}(2\times \pi \times \frac{i}{N})$ and $p_{n}{\left(i\right)}_{y}=l+R\times \text{sin}(2\times \pi \times \frac{i}{N})$. *h* and *v* denote the horizontal and vertical image dimensions. All computation are done on images that have been previously converted to gray scale images.

For the experiments conducted for this paper the score *var*(*R*,*N*)_*g *_is computed for 5 different radii (*R*=10:10:50) and the final result is averaged. This ensures that different texture frequencies are considered for the computation of the final score. This approach takes into consideration that the distribution of different tissue types is irregular as well.

### Experimental evaluation of optimal staining concentration

#### Experiments for lesion-to-background contrast

To evaluate the optimal staining concentration 2×2 cm patches of pig stomach mucosa were cut. To compute the optimal lesion-to-background score, *I*_*c*_, each time 10 patches were stained with a particular Indigo carmine concentration. Concentrations range from 0.2% to 1.6% in steps of 0.2%. Ten patches were left unstained. All patches were prepared with lesion imitations of type 0-IIc as described previously. Five out of the 10 patches stem from the fundus region and five from the pyloric region of the stomach. None of the patches stained with the same concentration were cut from the same stomach. In total, we examined 90 patches for these experiments: 5 patches per concentration and per stomach region and 9 different concentrations, including 10 unstained patches. The patches were placed in a glass container containing the respective dye concentration for 15 seconds and then washed with flowing lukewarm water for another 15 seconds. The patches were then photographed with a commercially-available industrial CCD camera (Point Grey Flea 2 FL2G-50S5M/C/ICX655 2/3 SuperHAD CCD, Point Grey Research Inc 12051 Riverside Way Richmond, BC, Canada). Such a scientific-quality and high-resolution apparatus decreases the amount of negative effects on the computation due to visual artifacts such as image distortion and sensor noise. It also allows for an accurate quantitative evaluation of the impact of different dye concentrations.

#### Experiments global texture variance

To evaluate the global texture variance score, *var*(*R*,*N*)_*g*_, experiments were conducted in the same way as for the lesion-to-background score. However, the patches this time were not prepared with lesion imitations. Furthermore, in this set of experiments the samples were stained with a Methylene blue concentration of 0.2% to 2.0% in steps of 0.2% and 0.2% to 1.6% in steps of 0.2% for fundus patches and pylorus patches respectively. 100 patches in total were examined in this evaluation.

#### Computation of scores and results

Pictures of 1400 x 1200 pixels were taken from each stained patch. These images were converted to gray-scale and the contrast score, *I*_*c*_, as well as the texture variance score, *var*(*R*,*N*)_*g*_, were computed for the respective patches.

### Ex-vivo Pig stomach experiments under magnetic guidance

To verify our results, the proposed modification of the preparation protocol and the newly determined optimal dye concentration were combined and tested on complete pig stomachs. In order to assess the effects of the proposed staining parameters, pig stomachs with lesion imitations were examined with magnetically guided capsule endoscopes, including lesion imitations, with and without applied staining. The pig stomachs were harvested fresh from a slaughterhouse with the esophagus and duodenum attached. 

1. Each pig stomach was prepared with either protruding (0-lp) lesions or non-protruding slightly depressed lesions of type 0-IIc. To prepare the stomach, first a 6 cm long cut in the fundus region was performed, the stomach was turned inside out using this cut and the artificial lesion imitations were applied. Eight lesion imitations of one type were distributed on all anatomical regions of the stomach. After applying the lesion imitations the stomach was turned back and the cut was stitched.

2. The modified preparation protocol previously described was applied to the stomach without the staining step M.2.

3. A capsule endoscope was introduced through the esophagus. The esophagus and duodenum were closed with clips to prevent water from running out of the stomach. The stomach was placed on a foam support with a stomach-shaped hole. This support maintained the natural shape of the stomach. The stomach was covered with a plastic sheet and the receiving antennas for the capsule endoscope were placed on this sheet.

4. The stomach was placed in the guidance magnet. In approximately 10 minutes all relevant anatomical regions of the stomach were visualized with the capsule endoscope.

5. The stomach was removed from the magnet and the water was flushed out.

6. The modified preparation protocol was again applied to the same stomach this time including the staining step M.2. For stomachs prepared with for 0-IIc lesion imitations, a 0.2% or 0.6% Indigo carmine solution was applied, depending on which anatomical section of the stomach the lesion was brought on. For the evaluation of general mucosa visibility and visibility of 0-lp lesions imitation 0.4% Methylene blue was applied.

7. Step 4 was repeated with the stained stomach. For better comparison of staining and unstained lesion imitation special emphasis was put on capturing lesion imitations from approximately the same viewpoint and viewing angle as with the unstained stomach. Lesion imitations of the same type applied to the same stomach were compared with and without staining from nearly the same viewpoint.

## Results

### Evaluation of an optimal dye concentration

**Figure 5:**
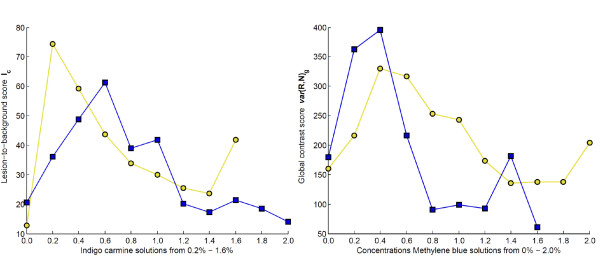
**Results of staining optimization: Left Figure: Yellow line with circle markers:*I***_***c ***_**score for the fundus stomach patches stained with Indigo carmine solutions from 0.2% - 1.6%.** Blue line with square markers: ***I***_***c ***_score for the pyloric stomach patches stained with indigo carmine solutions from 0.2% - 2.0%. Right Figure: Yellow line with circle markers: *var* (*R*,*N*)_*g*_ score for patches from the fundus stomach region, staining was performed with Methylene blue solutions from 0.2% - 2.0%. Blue line with square markers: *var* (*R*,*N*)_*g *_score for patches from the pylorus stomach region, staining was performed with Methylene blue solutions from 0.2% -1.6%.

In total 190 tissue samples have been examined to determine an optimal concentration of dye for the best lesion and mucosa visibility. Figure 5 summarizes these results. Each data point represents the score computed for one particular dye concentration averaged over five patches. The yellow line with circle markers (right figure) shows the texture variance score, *var*(*R*,*N*)_*g*_, for the Methylene blue concentration series from 0.0% to 2.0% with pig stomach patches stemming from the fundus region. A peak for the score appears at the 0.4% Methylene blue solution. The blue line with square markers shows the texture variance score, *var*(*R*,*N*)_*g*_, for the Methylene blue concentration series from 0.0% to 1.6% with pig stomach patches stemming from the pylorus region. A peak for the score occurs at the 0.4% Methylene blue solution.

The yellow line with circle markers (left figure) shows the lesions-to-background contrast score, *I*_*c*_, for the indigo carmine concentration series from 0.0% to 1.6% with pig stomach patches stemming from the fundus region. A peak for the score occurs at the 0.2% indigo carmine solution. The blue line with square markers shows the lesions-to-background contrast score, *I*_*c*_, for the indigo carmine concentration series from 0.0% to 2.0% with pig stomach patches stemming from the pylorus region. A peak for the score is formed at the 0.6% Indigo carmine solution.

Note that there is a 120% increase in the computed texture variance score, *var*(*R*,*N*)_*g*_, between the non stained tissue and the maximal score within the concentration series for the pylorus tissue sample and 105% increase for the fundus. For the lesions-to-background score, *I*_*c*_, we noted an increase of 205% for the pylorus and 469% for the fundus samples.

### Application on a optimal dye in an ex-vivo experiment

**Figure 6:**
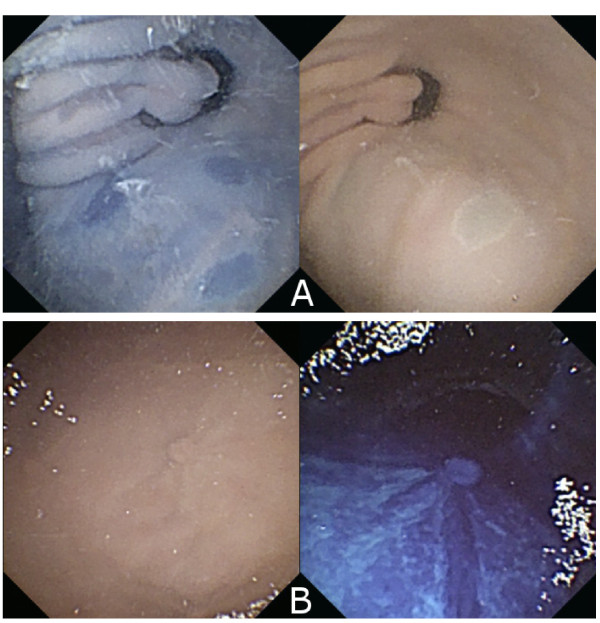
**Experiments in a pig stomach under magnetic guidance: Comparison of stained and unstained pig stomachs examined with a guided capsule endoscope.** Top: Lesion imitation of type 0-IIc with 0.3% Indigo carmine staining. (**A**) left: without staining, (**A**) right: with staining. Bottom: Lesion imitation of type 0-Ip with 0.4% Methylene blue staining. (**B**) left: without staining, (**B**) right: with staining.

Results from the previously conducted search for an optimal concentration were transferred to and applied to ex-vivo experiments with six pig stomachs prepared with lesion imitation as previously described. Visual inspection shows that the general mucosa visibility and the lesion visibility are enhanced through the staining procedure (See Figure 6). Quantitative analysis supports this observation. The lesions-to-background score *I*_*c*_ and the texture variance score *var*(*R*,*N*)_*g*_ were computed for the images stemming from the ex-vivo experiments. The texture variance score, *var*(*R*,*N*)_*g*_, in the pylorus region of the stomach was increased by 123% between the unstained and stained tissue patches compared to the 120% increase in the experimental pig stomach patches. The lesion-to-background score, *I*_*c*_, was increased by 95% between the unstained and stained lesions in the pylorus region and is, therefore, only half as significant compared to the experimental results computed for the pig stomach patches.

### Modification of preparation protocol

A modified preparation protocol for MGCE with chromoendoscopy was proposed. The protocol was tested in an ex-vivo pig stomach study under magnetic guidance of a capsule endoscope. No negative impact on the underwater visibility or steerability of the capsule could be observed.

## Discussion

### Staining procedure

The proposed modification to the preparation protocol did not have any negative effects on the examination in general. The visibility in the water-filled stomach was not disturbed by the additional stain applied during the modified protocol. This also holds true for the steerability of the capsule. Since the stain did not significantly change the viscosity of the water, the mechanical forces applied to the capsule by the magnetic fields were not affected either. Anatomical structures and landmarks (such as pylorus or cardia) are enhanced by the stain and can be easily spotted, making visual navigation easier for the operator. The darker appearance of the mucosa did not result in a underexposed and dark image in MGCE because the electronic shutter time adjustment of the capsule was able to correct for the dimmer setup. Application of water and dye for the proposed modified staining protocol has all been performed through the esophagus in order to simulate actual oral application. The oral application of dye for the examination of the stomach involving chromoendoscopy has been previously reported in humans.This suggests that the modified staining protocol is applicable to capsule examinations in the stomach in combination with MGCE.

### Evaluation of optimal stain concentration

190 pig stomach mucosa patches were stained with different staining concentrations and lesion imitations in order to systematically find an optimal staining concentration in the subsequent ex-vivo experiments. Compared to previous studies ([28,29]) objective criteria were established in order to determine the optimal concentrations. The experiments provide a clear result on the optimal staining concentration for different lesion types and different dye types. The staining concentration for an optimal lesion-to-background contrast differs when the dye (Indigo carmine) is applied to lesion imitations in different anatomical regions of the stomach. Those differences in optimal staining concentrations for different anatomical regions were previously observed in [28] due to differences in the cell epithelium. In our work, we also observe these differences for different stomach regions. Lesion imitations are applied to the pylorus and cardia region. Endocrine glands in the pylorus region produce additional mucus that dilutes the 10% HCL acid used for lesion imitations. The damage to the epithelium is therefore modest compared to the damage generated in the cardia region. A dye with higher concentration has, thus, a stronger effect on the staining of the damaged mucosa. The staining concentrations for the enhancement of global texture variance with Methylene blue is the same for both anatomical regions. As a vital stain, Methylene blue is absorbed by specific cell types. Since neck cells (isthmus of the gland) and enteroendocrine cells (base of the gland) are similar in both regions the optimal dye concentration is also the same.

### Ex-vivo pig stomach experiments

Optimal staining concentrations were transferred to ex-vivo experiments using a complete pig stomach under magnetic guidance. The lesion and mucosa visibility shows a similar behavior compared to the pig stomach tissue samples. The proposed methodology could, therefore, be further generalized for the assessment of dye concentration in capsule endoscopy and/or endoscopy in general. Furthermore, we showed that results of optimal staining concentration obtained with a high quality camera could be transfered to the ex-vivo stomach experiments where a capsule endoscope with a low resulution camera was used.

### Technical limitations of ex-vivo experiments

Although freshly resected pig stomachs were used for all experiments the staining characteristics might be different from in-vivo experiments. This also holds for the authenticity of lesion imitations.

### Future outcomes

Magnetically guided capsule endoscopes are an active field of research. The popular and established technique of chromoendoscopy may find its way to human MGCE procedures so that capsule examination and diagnosis can benefit from improved tissue localization and characterization.

## Conclusion

From the first ex-vivo experiments combining chromoendoscopy and magnetically guided capsule endoscopy we conclude that chromoendoscopy may be applied in MGCE and improves the mucosa and lesion visibility. Systematic evaluation provides important information on appropriate staining concentrations for MGCE examination with respect to different dyes and anatomical differentiation. However, further animal and human ex-vivo and in-vivo studies are necessary before transferring this technique into clinical trials.

## Abbreviations

MGCE: Magnetically guided capsule endoscopy; DOF: Degrees of freedom; EGD: Esophagogastroduodenoscopy; EGC: Early gastric cancer; lbp: Local binary pattern.

## Competing interests

• P. W. Mewes: PhD thesis financially supported by Siemens AG (Healthcare Sector), Employee Siemens AG (Healthcare Sector)

• S. Foertsch: PhD thesis financially supported by Siemens AG (Healthcare Sector)

• A. Lj. Juloski: Employee Siemens AG (Healthcare Sector)

• E. Angelopoulou: Salary: Spouse: Management Position Siemens AG (Healthcare Sector) Spouse: Ownership interest: Stock Shareholder Siemens AG (Healthcare Sector)

• A. Hornegger: Head of Pattern Recognition Lab: Joint projects with Siemens AG (Healthcare Sector)

• D. Guldi: The author disclosed no financial relationships relevant to this publication

• H. Messmann: The author disclosed no financial relationships relevant to this publication

• S.K. Goelder: The author disclosed no financial relationships relevant to this publication.

## Authors’ contributions

PWM conception and design of the study, analysis and interpretation of data, drafting of manuscript. SF study supervision, conception and design of the study. ALjJ critical revision of the manuscript for important intellectual content, drafting and revision of the manuscript, approval of the final version of the manuscript. EA critical revision of the manuscript for important intellectual content, drafting and revision of the manuscript, approval of the final version of the manuscript. SKG study supervision, drafting and revision of the manuscript, approval of the final version of the manuscript. DG approval of the final version of the manuscript. JH approval of the final version of the manuscript. HM study supervision, drafting and revision of the manuscript, approval of the final version of the manuscript. All authors read and approved the final manuscript.
